# Double burden of malnutrition at household level: A comparative study among Bangladesh, Nepal, Pakistan, and Myanmar

**DOI:** 10.1371/journal.pone.0221274

**Published:** 2019-08-16

**Authors:** Asibul Islam Anik, Md. Mosfequr Rahman, Md. Mostafizur Rahman, Md. Ismail Tareque, Md. Nuruzzaman Khan, M. Mahmudul Alam

**Affiliations:** 1 Department of Population Science and Human Resource Development, University of Rajshahi, Rajshahi, Bangladesh; 2 Department of Population Sciences, Jatiya Kabi Kazi Nazrul Islam University, Trishal, Mymensingh, Bangladesh; 3 Department of Statistics, University of Rajshahi, Rajshahi, Bangladesh; University of Dhaka, BANGLADESH

## Abstract

**Background:**

The coexistence of overweight mother and stunted child at the same household is a type of Double Burden of Malnutrition at Household Level (DBMHL). This particular public health concern is now emerging at an alarming rate among most of the South Asian and its neighboring lower-and-middle income countries which are going through nutritional transition. This study has examined the prevalence rate and the risk factors of DBMHL along with the socio-economic inequality in DBMHL among Bangladesh, Nepal, Pakistan, and Myanmar.

**Methods:**

Latest Demographic and Health Survey datasets were used in this study. To identify the significant association of DBMHL with socio-demographic characteristics, a multivariate technique named as logistic regression model, and for measuring socio-economic inequalities in DBMHL prevalence, relative index of inequality (RII) and slope index of inequality (SII) were used.

**Results:**

The prevalence rates of DBMHL were 4.10% (urban: 5.57%, rural: 3.51%), 1.54% (urban: 1.63%, rural: 1.42%), 3.93% (urban: 5.62%, rural: 3.20%), and 5.54% (urban: 6.16%, rural: 5.33%) respectively in Bangladesh, Nepal, Pakistan, and Myanmar. The risk ratios (RR) obtained from RII for Bangladesh, Nepal, Pakistan and Myanmar were 1.25, 1.25, 1.14, and 1.09, respectively, and β coefficient from SII were 0.01, 0.004, 0.005, and 0.006 unit respectively. In addition to not breastfeeding [Bangladesh (AOR: 1.55; 95% CI: 1.11–2.15), Myanmar (AOR: 1.74; 95% CI: 1.02–2.95)], respondent’s older age (in Bangladesh, Nepal, and Myanmar), child’s older age (in Pakistan and Myanmar), and middle and rich groups of wealth-index (in Bangladesh and Pakistan) were strong risk factors for DBMHL. On the other hand, female child [Nepal (AOR: 0.50; 95% CI: 0.26–0.95), Pakistan (AOR: 0.58; 95% CI: 0.41–0.84)], higher education [in Pakistan], respondent not participated in decision making [in Bangladesh and Nepal] and media access [Nepal (AOR: 0.44; 95% CI: 0.20–0.98)] had negative association with DBMHL.

**Conclusion:**

The DBMHL persists in all selected countries, with a higher prevalence in urban areas than in rural areas. In order to control the higher prevalence of DBMHL in urban areas, respective countries need urgent implementation of multisectoral actions through effective policies and empowering local communities.

## Introduction

Malnutrition, either under-nutrition or over-nutrition, is a persistent global public health concern which causes different types of diet-related non-communicable diseases [1,2]. Recently a report on global child malnutrition documented that most of the low and middle income countries are going through the nutrition transition, and experiencing the coexistence of dual faced malnutrition, i.e. maternal overweight and undernourished child in a family. However, the prevalence of stunting is declining too slowly while maternal overweight continues to rise globally [3]. Although there is a declining trend of child malnutrition in Bangladesh and its neighboring countries (such as Nepal, Pakistan, and Myanmar), the prevalence of child malnutrition is still high in these countries; whereas, overweight and obesity among women have significantly increased [4–10]. These countries are now facing a subtle problem named ‘double burden of malnutrition’, i.e., coexistence of undernutrition and obesity. This double burden of malnutrition has been observed at different level, i.e., country, household, and even individual level [11,12]. In the case of household level, double burden of malnutrition has been defined as the coexistence of stunted child along with overweight mother within the same household [13–18]. This definition of double burden of malnutrition at household level (DBMHL) is the one considered in the analysis. A survey conducted among 131 developing countries found that the magnitude of the coexistence of overweight-mother and undernourished-child pair at households were ranging from as low as 1.8% in Ethiopia to as high as 15.9% in Egypt [19]. At present, the prevalence of DBMHL in Bangladesh is reported to be approximately 4% [15,20].

The most denunciative outcome of DBMHL is that, DBMHL is an important promoter of double burden of diseases [21]. With the aim of achieving one of the Sustainable Development Goals (SDGs) 2.2 (ending all forms of malnutrition), most of the low-and-middle income countries are now focusing to reduce the terrible crisis of both DBMHL and double burden of disease [22]. Moreover, DBMHL confers a serious and negative economic impact on individuals and populations. For example, through its effects on health, DBMHL increases health-care costs of a country, reduces productivity and slows economic growth, which, in turn, can perpetuate a cycle of poverty and ill-health for the long run [23]. Therefore, understanding the factors associated with DBMHL in developing countries are crucial to develop interventions to improve maternal and child health as well as achieving the second goal of SDGs (SDG-2).

Most of the extant literature on DBMHL were limited to small-scale sample and provided little information [18,24–27]. Earlier literatures documented that maternal and child’s older age, sex and birth order of children, disease occurred to children, breastfeeding, working status of mothers, mother’s educational level, decision making autonomy, wealth-index, toilet facility of households, and respondent’s access to all media etc. had significant effect either on child malnutrition or maternal over-nutrition, or, on both [15,20,21,28–33]. This burden of malnutrition have serious developmental, economic, social and medical impacts on individuals as well their families and communities [23]. Such an important issue has hardly been studied in South Asian countries where the prevalence rates of maternal and child malnutrition are reported to be higher than the other regions of the world [34,35]. A nation-wide comparative study among some neighboring countries may depict clear amplitude of DBMHL in the corresponding regions. To the best of our knowledge, no study has been conducted in South Asian region that assessed the prevalence of DBMHL and the factors associated with it. To address this knowledge gap, this study has attempted to find the nation-wide prevalence of DBMHL and its associated risk factors in some selected countries in South Asian region, namely Bangladesh, Nepal, Pakistan and Myanmar.

## Methods

### Data

In this study, data for Bangladesh, Nepal, Pakistan and Myanmar was extracted from the latest Demographic and Health Surveys (DHS) of the respective countries. Since 1984, MEASURE DHS project has been providing assistance to conduct standardized household sample surveys in low-and-middle income countries with a special focus on health, socioeconomic, nutrition, and fertility-related information from women of reproductive age (15–49 years) [36]. After developing a rigorous area-based sampling design, the DHS program has employed multistage stratification and probabilistic sampling with each unit having a defined probability of selection [37]. In most of the included survey, sampling was stratified according to urban and rural areas, and furthermore in geographic or administrative regions. The DHS sampling framework and sample designs vary from country to country according to its population and other geographical factors [37]. In this study, information of households that have at least one mother-child pair from Bangladesh, Nepal, Pakistan, and Myanmar were used to identify the prevalence and risk factors of DBMHL. The procedure of selecting final sample size for selected countries has been shown in Table 1.

**Table 1:** Selection of sample size from the latest DHS of Bangladesh, Nepal, Pakistan and Myanmar.

	Country and DHS Year	Bangladesh2014	Nepal 2016	Pakistan 2012–13	Myanmar 2015–16
	Total Interviewed households	17300	11040	12943	12500
**Excluded Households**	*Households without at least one child-mother pair*	*9997*	*5571*	*5955*	*8447*
	*Households with flagged cases of maternal BMI and pregnant women*	*54*	*1366*	*167*	*50*
	*Households out of plausible limits of child stunting*	*677*	*1411*	*999*	*418*
	*Households with women who had twin births and whose children living elsewhere*	*36*	*5*	*13*	*16*
	*Missing information regarding covariates*	*58*	*17*	*39*	*28*
	Final Sample Size*	**6478**	**2670**	**5770**	**3541**

* Complete information of overall households with at least one child-mother pair.

Note: Exclusion criteria with numbers have been italicized.

### Outcome variable

According to World Health Organization (WHO), the double burden of malnutrition is the coexistence of undernutrition along with overweight, obesity or diet-related non-communicable diseases, within individuals, households and populations, and across the life-course [23]. In this study, double burden of malnutrition at household level (DBMHL), the outcome of interest, is defined as the coexistence of overweight mother and stunted child in the same household. A child was classified as stunted if his/her length/height was at least two standard deviations (< 2 SD) below the mean for their age [38]. Mother with body mass index more than or equal to 25 kg/m^2^ (BMI ≥ 25 kg/m^2^) was considered as overweight. A binary variable, DBMHL, was created where a household with an overweight mother and a stunted child was categorized as 1, and 0 if otherwise.

### Covariates

A variety of child and maternal socio-demographic characteristics that have been theoretically or empirically linked to child malnutrition [39–44] or maternal overweight [15,19,20,45–48] were included in the analysis. The children’s characteristics were age, sex, birth order, disease, and breast-feeding status. Age was classified as less than 24 months or 24 months and over. Sex was included because in South Asian Region, male children are valued more than female children, and receive preferential treatment, including better nutrition and care [49]. Birth order was classified as 1^st^ to 3^rd^ order, and 4^th^ order or more [15]. Dichotomous variable indicated whether a child was still breast-feeding, which is strongly associated with child health outcomes in developing countries [35], and whether he or she had suffered from any disease in the last 2 weeks. ‘Had any disease in the last 2 weeks’ indicates that, the children had suffered from either diarrhea or acute respiratory infection or fever in the last 2 weeks before the survey.

Maternal characteristics were age, education, work status, and decision making autonomy. Maternal age was classified as 15–24, 25–34 or 35–49 years. Education was categorized as no education, primary, secondary or higher. Work status was categorized as currently working or not. Maternal household decision-making autonomy was included because earlier literature suggests that enhancing maternal autonomy may be an important intervention for improving maternal and child nutrition [50]. In this study, maternal household decision-making autonomy was measured using mother’s responses to three questions that asked who makes decisions in household regarding obtaining health care for herself, making large purchases, and visiting family and relatives. Response categories were the respondent alone, the respondent and her husband, the respondent and someone other than her husband, her husband alone, someone else or other. For each of the three questions, a value of 1 was assigned if the respondent was involved in making the decision, and 0 if she was not, the values were summed and dichotomized as participated and not participated.

Finally, several household characteristics were included in the analyses. These include wealth-index, toilet facility, place of residence (urban or rural), and media exposure (watching TV, listening radio, and reading newspapers). The wealth-index was calculated as described in the corresponding country’s latest and available DHS reports [8], using principal component analysis of the assets owned by households in urban and rural areas [51,52]. The score was divided into five equal quintiles with the first, representing the poorest 20%, and the fifth, representing the richest 20% [53,54]. The other measures indicated whether the household had sanitary toilet facilities and some degree of media access. For the types of toilet facility, ‘Improved toilet’ refers toilets with flush systems, VIP latrine, pit latrine with slab, and open pit latrine; and ‘Not improved’ refers toilets with no facility/bush, field, bucket toilet, and hanging toilets. Media access refers to those households where there was access to watching TV, listening radio, and reading newspaper at least once a week. For each of the two categories of media access, a value of 1 was assigned if there was access to media, and 0 if there was not.

### Statistical analysis

We have calculated the prevalence of DBMHL for Bangladesh, Nepal, Pakistan and Myanmar. Chi-square tests were used to identify the bivariate association between various socio-demographic variables and DBMHL. To measure different direction of socioeconomic inequalities in DBMHL, two regression based methods were used: (i) relative index of inequality (RII); (ii) slope index of inequality (SII). These two measures of inequality were used because the RII and SII took into account the prevalence of DBMHL across the whole socioeconomic distribution in the study population of each country [55]. To compute SII and RII, linear regression and modified poisson approach were used [56]. A positive value of SII means the increment of health indicator with increasing socio-economic status (SES). Again, values of RII < 1 indicate that the poor are more risky to be exposed to adverse SES outcomes compared with the rich. Finally, multivariate logistic regression models were used to assess the association between DBMHL and socio-demographic variables; adjusted odds ratios with 95% confidence interval were estimated to assess the strength of the association of DBMHL with predictor and covariates. In all analyses, the significant level was set at p < 0.05. All the analyses were performed using Stata’s ‘SVY’ command in order to control the effect of the complex survey design. To evaluate the possible collinearity, we used variance inflation factor and found no multicollinearity problem among variables. Stata version 14.2 [57] was used to carry out all the analyses.

### Ethical consideration

The surveys were approved by Macro-institutional review board; and the survey protocols were reviewed and approved by National Research Ethics Committee of the respective country [58]. For individual and household interview, verbal informed consent was obtained from the respondents. For anthropometric measurements, informed consent was also obtained after the individual interview. Ethical approvals were obtained by DHS and respective countries prior to the data collections.

## Results

### The prevalence of DBMHL

**Fig 1:**
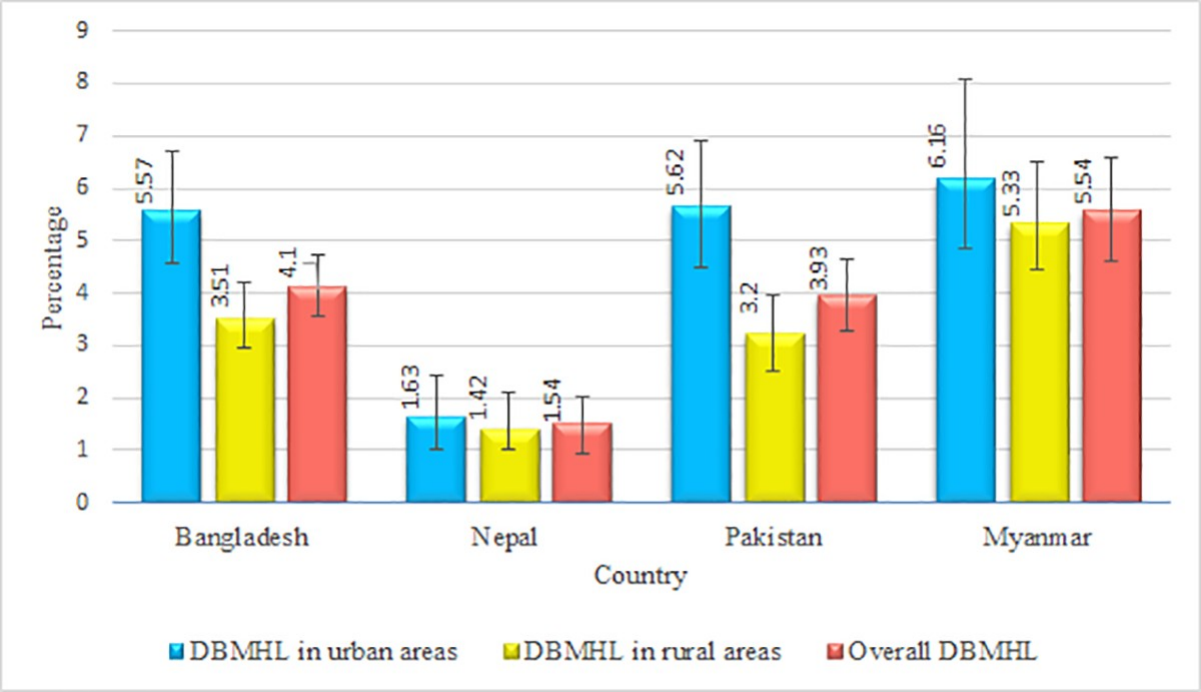
Prevalence of DBMHL in Bangladesh, Nepal, Pakistan and Myanmar. Note: Total observation in Bangladesh, Nepal, Pakistan, and Myanmar was 6478 (rural: 4399, urban: 2079), 2670 (rural: 1144, urban: 1526), 5770 (rural: 3168, urban: 2602), and 3541 (rural: 2728, urban: 813) respectively.

From Fig 1, the highest prevalence of DBMHL was observed in Myanmar (5.54%) and the lowest prevalence was in Nepal (1.54%). The prevalence of DBMHL in Bangladesh was 4.10% and in Pakistan 3.93%. This prevalence was found to be higher in urban areas than in rural areas among these four countries.

### Background characteristics

**Fig 2:**
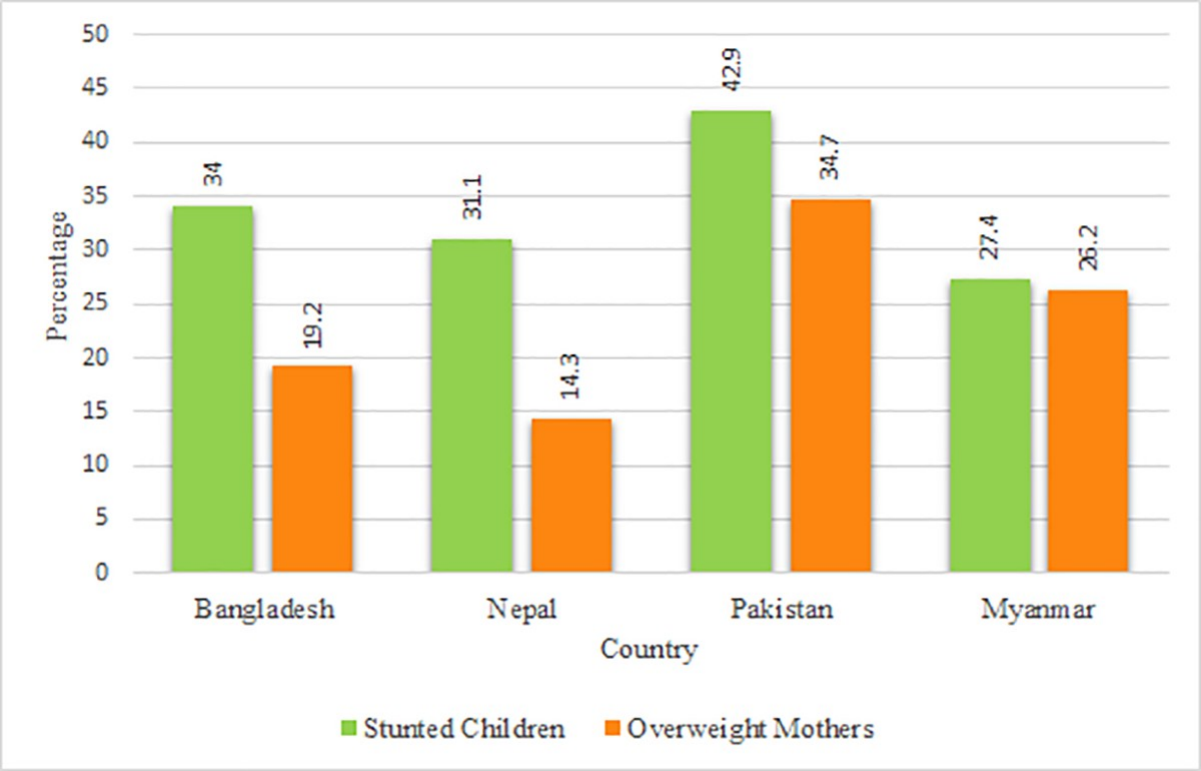
Percentages of households with stunted children and overweight mothers. Note: Percentages were weighted; the total number of households of Bangladesh, Nepal, Pakistan, and Myanmar were 6478, 2670, 5770, and 3541 respectively.

The percentages of households with stunted children and overweight mothers are presented in Fig 2. In Bangladesh, 34% stunted children and 19% overweight mothers were found at household level. The prevalence rates of stunted children and overweight mothers at household level were higher in Pakistan than other countries.

Table 2 shows the background characteristics of the selected households in Bangladesh, Nepal, Myanmar, and Pakistan. The prevalence of younger children (0 to 23 months) was the highest (72%) in Nepal. More than half of the children had suffered from diseases (either diarrhea or acute respiratory infection or fever) in the last 2 weeks before the survey, and they were currently breastfed. But, the prevalence of practicing breastfeeding was significantly the highest (93%) in Nepal. Almost half of the mothers were aged below 25 years in Bangladesh and Nepal, however, in Myanmar and Pakistan most of the mothers were aged between 25–34 years. Again, more than half of the respondents were not currently working (except Myanmar), and were not participating in making household decisions.

**Table 2:** Basic characteristics of the households with at least one child-mother pair in Bangladesh, Nepal, Myanmar, and Pakistan.

Basic characteristics	Bangladesh	Nepal	Pakistan	Myanmar
	N (%)^a^	N (%)^a^	N (%)^a^	N (%)^a^
**Child’s age**				
0 to 23 months	2914 (45.77)	1910 (71.98)	3093 (54.98)	1733 (47.01)
24 to 59 months	3564 (54.23)	760 (28.02)	2677 (45.02)	1808 (52.99)
**Had any disease in the last 2 weeks**				
No	2242 (34.27)	851 (33.02)	2501 (40.34)	1403 (43.81)
Yes	4236 (65.73)	1819 (66.98)	3269 (59.66)	2138 (56.19)
**Birth order**				
1–3	5480 (84.72)	2257 (84.90)	2958 (51.32)	2596 (77.51)
≥ 4	998 (15.28)	413 (15.10)	2812 (48.68)	945 (22.49)
**Sex of child**				
Male	3382 (52.74)	1454 (53.45)	2984 (52.52)	1845 (51.21)
Female	3096 (47.26)	1216 (46.55)	2786 (47.48)	1696 (48.79)
**Respondent’s age**				
15–24	3134 (48.49)	1322 (48.40)	1126 (20.08)	655 (18.26)
25–34	2812 (43.77)	1191 (45.75)	3132 (55.13)	1797 (51.77)
35–49	532 (7.74)	157 (5.85)	1512 (24.79)	1089 (29.96)
**Currently breastfeeding**				
Yes	3657 (56.68)	2495 (92.78)	2835 (50.79)	1956 (54.91)
No	2821 (43.32)	175 (7.22)	2935 (49.21)	1585 (45.09)
**Highest educational level**				
No education	953 (15.58)	761 (29.67)	3187 (56.92)	548 (15.50)
Primary	1749 (27.26)	514 (19.94)	794 (15.91)	1559 (45.93)
Secondary	3046 (47.30)	969 (35.00)	1035 (17.55)	1145 (29.98)
Higher	730 (9.86)	426 (15.39)	754 (9.61)	289 (8.59)
**Work-status**				
Not working	4893 (74.45)	1364 (53.81)	4659 (73.76)	1621 (43.15)
Working	1585 (25.55)	1306 (46.19)	1111 (26.24)	1920 (56.85)
**Wealth-index**				
Richest	1317 (19.84)	340 (14.68)	1162 (16.69)	503 (15.65)
Richer	1332 (20.32)	508 (20.43)	1070 (18.43)	643 (18.25)
Middle	1268 (20.05)	552 (21.99)	1061 (18.97)	644 (17.36)
Poorer	1216 (18.77)	580 (21.42)	1159 (21.49)	778 (21.89)
Poorest	1345 (21.03)	690 (21.48)	1318 (24.43)	973 (26.86)
**Media access**				
No	2400 (37.13)	569 (22.50)	3141 (54.56)	615 (17.29)
Yes	4078 (62.87)	2101 (77.50)	2613 (45.44)	2926 (82.71)
**Household decision making autonomy**				
Respondent participated	2778 (45.81)	658 (25.96)	2541 (48.42)	2308 (64.70)
Not participated	3700 (54.19)	2012 (74.04)	3229 (51.58)	1233 (35.30)
**Types of toilet facility**				
Improved	5557 (85.27)	2023 (71.44)	4559 (70.16)	2831 (80.63)
Not improved	921 (14.73)	647 (28.56)	1211 (29.84)	710 (19.37)
**Place of residence**				
Urban	2079 (25.82)	1526 (53.92)	2602 (30.26)	813 (22.98)
Rural	4399 (74.18)	1144 (46.08)	3168 (69.74)	2728 (77.02)
**Total**	**6478**	**2670**	**5770**	**3541**

N, Number of observation

^a^ Percentages are weighted.

### Association of DBMHL with socio-demographic characteristics

Table 3 presents the results of association of DBMHL with selected socio-demographics in Bangladesh, Nepal, Pakistan, and Myanmar. The proportion of DBMHL was higher among older children in Bangladesh (4.9% vs 3.0%), Nepal (2.4% vs 1.2%), Pakistan (6.1% vs 2.2%), and Myanmar (8.3% vs 2.4%). The households with no breastfeeding practice have higher proportion of DBMH than the households with breastfeeding practice; e.g., Bangladesh (5.4% vs 3.0%), Nepal (3.2% vs 1.4%), Pakistan (5.7% vs 2.2%), and Myanmar (8.5% vs 3.3%).

**Table 3:** Bivariate association (percentage) and adjusted associations (odds ratio) between different socio-demographic determinants and the DBMHL for the respective countries.

Determinants	Bangladesh (n = 6478)		Nepal (n = 2670)		Pakistan (n = 5770)		Myanmar (n = 3541)	
	(%)^a^	AOR (95% CI)^b^	(%)^a^	AOR (95% CI)^b^	(%)^a^	AOR (95% CI)^b^	(%)^a^	AOR (95% CI)^b^
**Breastfeeding**								
Yes	3.00	1 (Ref)	1.40	1 (Ref)	2.20	1 (Ref)	3.00	1 (Ref)
No	5.40	1.55* (1.11–2.15)	3.20	1.93 (0.64–5.85)	5.70	1.46 (0.85–2.51)	8.50	1.74* (1.02–2.95)
P-value (χ^2^)	P = 0.001		P = 0.067		P < 0.001		P < 0.001	
**Respondent’s age**								
15–24	2.70	1 (Ref)	1.20	1 (Ref)	1.80	1 (Ref)	2.90	1 (Ref)
25–34	5.30	1.83** (1.19–2.82)	1.60	1.27 (0.63–2.57)	3.70	1.71 (0.89–3.30)	4.80	1.22 (0.62–2.39)
35–49	5.50	2.01* (1.06–3.81)	3.70	3.63 (0.93–14.11)	6.30	2.64** (1.27–5.52)	8.30	2.03* (1.04–4.11)
P-value (χ^2^)	P < 0.001		P = 0.054		P < 0.001		P = 0.001	
**Child’s age**								
0 to 23 months	3.00	1 (Ref)	1.20	1 (Ref)	2.20	1 (Ref)	2.40	1 (Ref)
24 to 59 months	4.90	1.13 (0.72–1.76)	2.40	1.59 (0.76–3.32)	6.10	1.92** (1.18–3.11)	8.30	2.12* (1.16–3.83)
P-value (χ^2^)	P = 0.021		P = 0.042		P < 0.001		P < 0.001	
**Had disease in the last 2 weeks**								
No	4.30	1 (Ref)	0.90	1 (Ref)	4.10	1 (Ref)	4.40	1 (Ref)
Yes	3.90	0.81 (0.55–1.16)	1.90	1.74 (0.79–3.83)	3.80	0.99 (0.69–1.44)	6.40	1.05 (0.71–1.54)
P-value (χ^2^)	P = 0.608		P = 0.044		P = 0.627		P = 0.054	
**Sex of child**								
Male	3.70	1 (Ref)	2.00	1 (Ref)	4.80	1 (Ref)	5.10	1 (Ref)
Female	4.40	1.16 (0.82–1.65)	1.00	0.50* (0.26–0.95)	3.00	0.58** (0.41–0.84)	5.90	1.15 (0.82–1.58)
P-value (χ^2^)	P = 0.374		P = 0.032		P = 0.004		P = 0.343	
**Birth order**								
1–3	3.90	1 (Ref)	1.50	1 (Ref)	3.30	1 (Ref)	5.40	1 (Ref)
≥ 4	4.80	1.17 (0.76–1.81)	1.60	0.97 (0.35–2.70)	4.60	0.98 (0.63–1.53)	5.90	0.91 (0.57–1.46)
P-value (χ^2^)	P = 0.282		P = 0.864		P = 0.056		P = 0.616	
**Work status of Mother**								
Not working	4.20	1 (Ref)	1.40	1 (Ref)	4.00	1 (Ref)	4.30	1 (Ref)
Working	3.80	0.89 (0.62–1.27)	1.70	1.16 (0.57–2.36)	3.60	0.92 (0.60–1.41)	6.50	1.22 (0.79–1.86)
P-value (χ^2^)	P = 0.705		P = 0.654		P = 0.546		P = 0.035	
**Educational status of mother**								
No education	3.50	1 (Ref)	1.10	1 (Ref)	3.80	1 (Ref)	3.60	1 (Ref)
Primary	3.80	1.16 (0.76–1.77)	1.40	2.04 (0.73–5.72)	4.60	0.92 (0.53–1.57)	6.70	1.66 (0.94–2.91)
Secondary	4.20	1.08 (0.68–1.75)	2.10	4.44**(1.52–12.91)	4.30	0.63 (0.34–1.16)	4.50	1.10 (0.53–2.26)
Higher	4.60	0.93 (0.53–1.63)	1.50	2.31 (0.56–9.52)	3.00	0.35** (0.16–0.77)	6.20	1.33 (0.51–3.43)
P-value (χ^2^)	P = 0.726		P = 0.251		P = 0.591		P = 0.075	
**Wealth-index**								
Poorest	1.90	1 (Ref)	1.50	1 (Ref)	2.50	1 (Ref)	4.20	1 (Ref)
Poorer	2.80	1.49 (0.86–2.58)	0.60	0.40 (0.09–1.78)	2.90	1.20 (0.63–2.24)	5.10	1.09 (0.63–1.88)
Middle	5.90	2.90***(1.59–5.27)	1.70	1.43 (0.47–4.38)	4.00	1.58 (0.72–3.44)	8.40	1.73 (0.97–3.10)
Richer	4.30	1.87* (1.06–3.25)	2.20	1.58 (0.50–4.98)	5.70	2.48* (1.16–5.30)	4.70	0.89 (0.45–1.76)
Richest	5.40	2.02* (1.08–3.82)	1.80	1.11 (0.28–4.30)	5.30	2.59* (1.07–6.26)	6.00	1.12 (0.44–2.87)
P-value (χ^2^)	P < 0.001		P = 0.385		P = 0.007		P = 0.051	
**Household Decision making autonomy**								
Respondent Participated	4.90	1 (Ref)	3.10	1 (Ref)	4.10	1 (Ref)	5.80	1 (Ref)
Not participated	3.30	0.73* (0.55–0.98)	1.00	0.34** (0.16–0.71)	3.80	1.02 (0.69–1.50)	5.10	0.91 (0.60–1.37)
P-value (χ^2^)	P = 0.005		P = 0.001		P = 0.664		P = 0.511	
**Toilet Facility**								
Improved	4.00	1 (Ref)	1.80	1 (Ref)	4.50	1 (Ref)	5.90	1 (Ref)
Not improved	4.40	1.52 (0.73–3.12)	1.00	0.72 (0.25–2.04)	2.70	1.02 (0.60–1.71)	3.80	0.83 (0.47–1.45)
P-value (χ^2^)	P = 0.779		P = 0.218		P = 0.018		P = 0.071	
**Media Access**								
No	2.60	1 (Ref)	1.80	1 (Ref)	3.80	1 (Ref)	3.60	1 (Ref)
Yes	4.90	1.48 (0.98–2.23)	1.50	0.44* (0.20–0.98)	4.10	1.03 (0.71–1.48)	5.90	1.40 (0.82–2.39)
P-value (χ^2^)	P = 0.001		P = 0.553		P = 0.682		P = 0.041	
**Place of residence**								
Urban	5.60	1 (Ref)	1.60	1 (Ref)	5.60	1 (Ref)	6.20	1 (Ref)
Rural	3.50	0.73 (0.53–1.01)	1.40	1.16 (0.52–2.60)	3.20	0.72 (0.45–1.18)	5.30	0.82 (0.46–1.48)
P-value (χ^2^)	P = 0.002		P = 0.727		P = 0.003		P = 0.495	

Ref., reference; AOR, Adjusted Odds Ratio; n, total observation; P-value (χ^2^), P-value obtained from chi-square estimation

^a^Prevalence of DBMHL in percentage obtained from chi-square estimation

^b^95% confidence intervals

Level of significance

*p<0.05

**p<0.01

***p<0.001

Not breastfed children had 1.55 and 1.74 times higher odds of suffering from DBMHL than the breastfed children in Bangladesh and Myanmar, after adjusting for all covariates. Surprisingly, female child were negatively associated with DBMHL in Nepal [AOR, 0.50; 95% CI, 0.26–0.95] and Pakistan [AOR, 0.58; 95% CI, 0.41–0.84]. Compared to poorest households, Bangladeshi rich (both richer and richest) and middle income households were experiencing higher DBMHL, and in Pakistan, only the rich (both richer and richest) had experienced it. However, secondary educated mothers (only in Nepal), older maternal age (except Nepal), and older children (except Bangladesh and Nepal) had significant and positive association with DBMHL. Respondent who did not participate in making household decision (in Bangladesh and Nepal) and media access (only in Nepal) was less likely to be associated with DBMHL.

### Summary measures of SES inequality

Table 4 shows summary measures of SES inequality in DBMHL for the selected countries. Significant RII for Bangladesh, Nepal, and Pakistan were 1.25, 1.25, and 1.14 respectively, indicating that a move from the poorest to the richest of the SES distribution was associated with a 25%, 25%, and 14% increase in DBMHL in Bangladesh, Nepal, and Pakistan. And, SII indicated that one unit change from the poorest to the richest of the SES group was associated with 0.01, 0.004, and 0.005 unit increase in DBMHL in Bangladesh, Nepal, and Pakistan, respectively.

**Table 4:** Summary measures of Socio-economic status (SES) inequality in DBMHL in Bangladesh, Nepal, Myanmar, and Pakistan.

Country (n)	Relative index of inequality (RII) RR (95% CI)	Slope index of inequality (SII) β coefficient (95% CI)
**Bangladesh (n = 6478)**	1.25 (1.16,1.37)	0.0100 (0.0070,0.0130)
**Nepal (n = 2670)**	1.25 (1.01,1.54)	0.0040 (0.0002,0.0070)
**Pakistan (n = 5770)**	1.14 (1.04,1.24)	0.0050 (0.0020,0.0070)
**Myanmar (n = 3541)**	1.09 (0.99,1.20)	0.0060 (-0.0001,0.0110)

n, total number of observation

## Discussion

This study has investigated and compared the amplitude of the DBMHL, and the significant risk factors those are accountable for occurring DBMHL especially the socio-economic inequality in Bangladesh, Nepal, Pakistan, and Myanmar. It is documented from this study that the current prevalence rate of DBMHL is fairly high in these countries. The current prevalence rate of DBMHL in the African, Asian, and Latin American countries are as follows: Ethiopia (1.8%), Senegal (3%), Chad (3.5%), Uganda (3.6%), Tanzania (4.1%), Rwanda (4.4%), Kazakhstan (2.5%), Vietnam (5%), Uzbekistan (4.1%), Jordan (3.6%), Cambodia (4%), and Columbia (4%) [12,19]. Compared to the above countries, the prevalence of DBMHL is higher in Myanmar (5.54%). It is noteworthy that, the prevalence of overall DBMHL in Bangladesh (4.10%) is almost the same as the earlier estimates [14,15,20]. Moreover, our study has revealed that DBMHL in Bangladesh is higher than the other South Asian countries, Nepal (1.54%) and Pakistan (3.93%); where the previous literatures have supported it too [20,59].

Our study has shown that, there is a positive association between higher wealth-index of the households and DBMHL, especially in Bangladesh and Pakistan. Some other studies have also supported our result by revealing that, child malnutrition and maternal overweight were highly observed among middle income families [60,61]. The positive association between higher wealth-index and DBMHL are in line with one of the previous studies conducted in Bangladesh and its neighboring countries [15], but appears to be contradicted with the studies from Latin American countries, where the prevalence of DBMHL is more common in lower wealth-index groups. In South Asian region, such contrast can be due to the fact that, practices like- excessive intake of processed energy-dense foods, lack of physical exercise, and soft drinks consumptions are more common in middle and high (richer and richest) income households [62].

We have found that, older mothers were more associated with the increased risk of DBMHL than the younger mothers in the study countries except Nepal. This is in a line with other studies that showed women aged 30 years or older were overweight and obese in Bangladesh, Myanmar, and Pakistan [4,9,63,64]. Because of sedentary lifestyle and reduction of metabolic rates, obesity is increasing with age among women [65]. Following this issue, among Myanmar and Bangladeshi households, our findings indicated that, not breastfeeding mothers showed positive association with DBMHL than those continuously breastfed their children. Breastfeeding burns extra calories of mothers; so, long term breastfeeding not only helps mothers to lose pregnancy weight faster, but also provides all essential micronutrients a baby needs [47,66]. Thus, poor breastfeeding practices can contribute to both maternal overweight and child malnutrition. Though the breastfeeding has become universal in almost all Asian countries with extended median duration of breastfeeding, surprisingly our study has revealed that, among the children aged 24 to 59 months old, Myanmar and Pakistani households are facing more DBMHL significantly than the 0 to 23 months old children. As breastfeeding has a protective effect on both stunting in children and overweight in mothers, it is plausible to have higher rates of DBMHL among children 24–59 months when breastfeeding has a low impact on child’s dietary adequacy and is less practiced by mothers. Recent studies have supported this outcome by investigating that, along with Pakistan, other countries of Asia are experiencing malnutrition among older children because of bypassing continuous breastfeeding practices [39,42,67,68]. The latest DHS report of Pakistan brought out that, only 1% children (18 to 23 months) were continuing breastfeeding and other complementary foods, and no information was found regarding breastfeeding practice among older children [9]. Such malnutrition among older children in Pakistan and Myanmar is dangerously shaping DBMHL. In our study, the prevalence of overall DBMHL in Nepal was comparatively lower than other countries; and it is partly due to higher prevalence of practicing breastfeeding mother and of younger children (0 to 23 months) in Nepal.

Female children of Nepal and Pakistani households have exhibited less association with DBMHL significantly than the male children. The prevalence of exclusive breastfeeding was higher among female children than male in both Pakistan and Nepal, which probably decreased DBMHL of the households with female children [7,9].

The Nepalese households with access to media showed significant negative association with DBMHL. Several studies conducted in Indian Subcontinent revealed that, children whose mothers did not have any access to mass media (i.e., newspapers, radio & TV) possessed higher probability of having severe and moderate malnutrition [69,70]. It was also found that, exposure to media increased the likelihood that mothers gave complementary foods and offered it at least twice daily with increased meal frequency and diversity [71]. On the other hand, at present, most of the countries have launched different types of media campaign to dispense child nutrition information and to reduce the consumption of oily and sugar-sweetened beverages in homes as a strategy to combat both malnutrition and obesity [71,72]. As, in Nepal, more than one-third households had access to media, and it is plausible to consider media access as one of the important protective factors against DBMHL in Nepal.

This study found a mixed result of the prevalence of DBMHL according to maternal education. While higher maternal education were positively associated with DBMHL in Pakistan, the Nepalese sample shows a negative impact of higher education on DBMHL in household. A similar study conducted in Myanmar reported that, among the women with secondary education, about 28% were overweight and 14% were obese [4]. Another study from Nepal [73] reported that, most of the educated mothers, who entered into employment had higher chance of being overweight than the general population; and, more than half of the population consider ‘being overweight’ as a symbol of ‘prosperity’. This could possibly explain the linkage between secondary educated mothers and increased DBMHL in Pakistan.

According to WHO [23], the DBMHL can be seen as a dual nutrition challenge for any country; addressing this DBMHL and its risk factors will be one of the major priorities in achieving SDG-2. The indicator 2.2 of the SDG-2 has pointed out to end all forms of malnutrition by 2030. Aiming this issue, this study has brought out the prevalence rates of DBMHL and the underlying determinants of DBMHL of the selected countries. If the risk factors of DBMHL, obtained from our study, can be averted by the respective countries with proper interventions, programmes, and policies, the prevalence of malnutrition of mother-child pair can be checked. And eventually, the way of achieving SDG-2 will be smoother for those countries.

This study has several strengths and limitations. The main strengths are: it is based on nationally representative surveys with large sample sizes and low percentages of missing information which provide sufficient statistical power to give more reliable and unalloyed results with greater precision and power. The strengths of the DHS data for studying population health of any country include very high response rates, national coverage, standard data collection procedures, and interviewer training [74–76]. However, because of cross-sectional nature of our data, causality cannot be suspected properly. This study is also unable to include other apparently important variables concerning DBMHL such as daily dietary pattern, physical activity, caregiving practices, cultural influences, pregnancy and birth information etc. due to unavailability of information in the DHS. Finally, we have used BMI, to assess the nutritional status of mother. However, the use of standard WHO definitions for overweight and obesity may have introduced bias since they do not consider the ethnic and region specific recommendations for BMI cut off values for overweight and obesity [77]; because, some of the mothers, still at risk for overweight and obesity from lower BMI values, may have been overlooked.

## Conclusion

This is the first study where the overall diversity and the risk factors of DBMHL were separately and comparatively identified for Bangladesh, Nepal, Pakistan, and Myanmar. The overall results suggested that maternal and child age as well as poor breastfeeding were risk factors for DBMHL with varying risk factors among countries; such as- secondary education of mothers was the risk factor for DBMHL in Nepal, whereas, higher education was the protective factor in Pakistan. Again, richer and richest wealth-index were found to be positively associated with DBMHL in Bangladesh and Pakistan. Media access at least once a week showed negative association with DBMHL in Nepal. In such complex circumstances, for introducing effective long-term recommendations and proactive actions against DBMHL, further studies have to be conducted by respective countries. Even though, simultaneously some actions along with the existing policies can be taken to reduce this burden, such as- providing education on the importance of equilibrium between energy intake and expenditure; promoting and supporting breastfeeding; ensuring conditions for optimal fetal and early child development; ensuring access to optimal maternal and antenatal nutrition and care; raising awareness among the rich people by highlighting the benefits of physical exercises; advertising campaigns through mass media on nutritional issues for sound feeding practices; and reducing poverty through empowering local communities. Finally, in order to achieve SDG-2, most of the Asian countries need to pay heed upon the risk factors of DBMHL to inhibit the spread of DBMHL.

## Abbreviations

AOR: Adjusted Odds Ratio
BDHS: Bangladesh Demographic and Health Survey
BMI: Body Mass Index
CI: Confidence Interval
DBMHL: Double Burden of Malnutrition at Household Level
DHS: Demographic and Health Survey
MDHS: Myanmar Demographic and Health Survey
NDHS: Nepal Demographic and Health Survey
PDHS: Pakistan Demographic and Health Survey
RII: Relative Index of Inequality
RR: Risk Ratio
SDG: Sustainable Development Goal
SES: Socio-Economic Status
SII: Slope Index of Inequality
WHO: World Health Organization
